# A New Quantitative Metric for Precise Classification of Diabetic Podocyte Injury Using Scanning Electron Microscopy

**DOI:** 10.1093/mam/ozaf122

**Published:** 2025-12-10

**Authors:** Faith Rooney, Chantal Allamargot, Jillian Williquett, Hua Sun

**Affiliations:** Division of Nephrology, Stead Family Department of Pediatrics, Carver College of Medicine, The University of Iowa, 200 Hawkins Dr, Iowa City, IA 52242, USA; Central Microscopy Research Facility, Office of the University of Iowa Vice President for Research, 200 Hawkins Drive, Iowa City, IA 52242, USA; Division of Nephrology, Stead Family Department of Pediatrics, Carver College of Medicine, The University of Iowa, 200 Hawkins Dr, Iowa City, IA 52242, USA; Division of Nephrology, Stead Family Department of Pediatrics, Carver College of Medicine, The University of Iowa, 200 Hawkins Dr, Iowa City, IA 52242, USA

**Keywords:** podocyte, scanning electron microscope, slit diaphragm

## Abstract

Podocytes are highly interdigitated epithelial cells in the glomerulus that maintain the kidney's filtration barrier, and their injury underlies the progression of diabetic kidney disease. Early podocyte damage is challenging to detect using light or transmission electron microscopy; new techniques like super-resolution microscopy remain limited in capturing the three-dimensional topography of podocytes. Scanning electron microscopy (SEM) offers superior spatial resolution and surface detail; however, standardized quantitative methods to analyze podocyte ultrastructure are lacking. In this study, we developed and compared three analytical approaches to quantify podocyte injury from SEM images in a streptozotocin-induced diabetic mouse model. Using ImageJ software, we measured the slit diaphragm (SD) fraction via (1) thresholding, (2) ridge detection, and (3) foot process plot profiling, comparing diabetic and nondiabetic podocytes. The ridge detection method showed the best diagnostic accuracy (88% sensitivity and 93% specificity), successfully distinguishing diabetic from healthy podocytes. Furthermore, SD fraction measurements correlated negatively with biomarkers of podocyte dysfunction and diabetic stress. This work establishes the first reliable, quantitative pipeline for detecting subtle early podocyte injury in diabetic kidney disease using SEM, providing a valuable tool for future mechanistic and therapeutic studies.

## Introduction

**Fig. 1.**
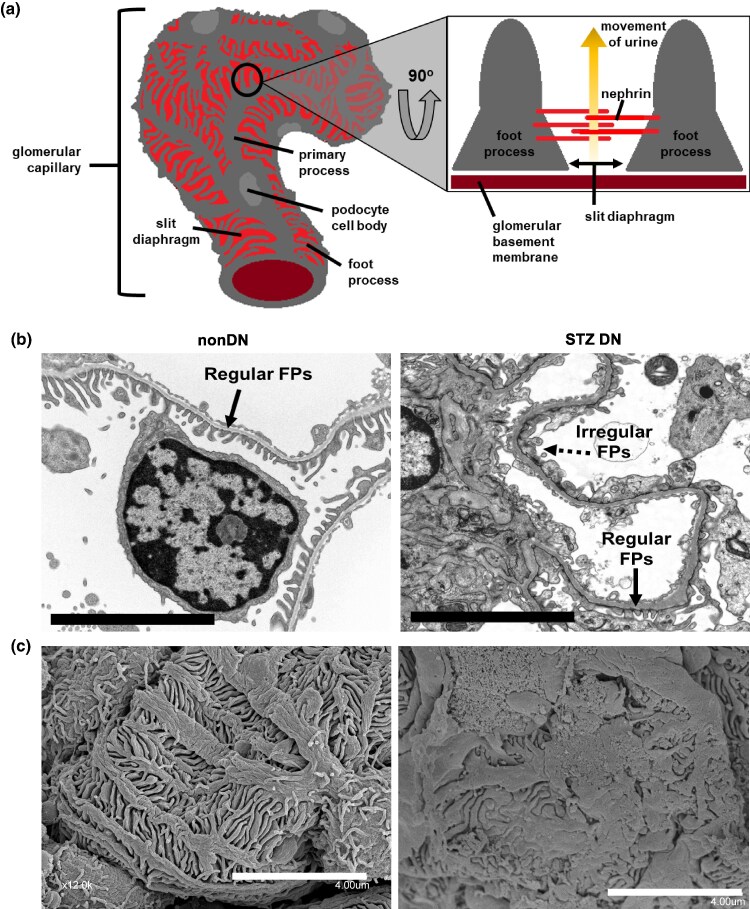
Glomerular ultrastructure. (**a**) Schematic illustration of the slit diaphragm (SD) area located between adjacent foot processes (FPs) on a glomerular capillary, highlighting the region targeted for quantitative measurement. Representative transmission electron microscopy (TEM) images (**b**: Scale bar = 5 *μ*m) and scanning electron microscopy (SEM) images (**c**. Scale bar = 4 *μ*m) comparing glomerular ultrastructure in nondiabetic and diabetic mice.

Podocytes are highly specialized epithelial cells that wrap around glomerular capillaries with intricately interdigitated foot processes (FPs) (Kawachi & Fukusumi, 2020). The slit diaphragm (SD), a tight junction-like structure located between FPs, is composed of nephrin and other transmembrane proteins that form a molecular sieve within the glomerular filtration barrier (Fig. 1a). This structure prevents the leakage of circulating proteins into the urine as it passes into Bowman's capsule (Martin & Jones, 2018). Thus, microalbuminuria, the excessive leakage of albumin into the urine, is typically an indicator of podocyte dysfunction. The podocyte is continuously exposed to hemodynamic and metabolic stressors within the glomerulus, and this exposure can lead to a spectrum of cellular injuries—from foot process effacement to detachment and cell death—culminating in glomerulosclerosis. Our lab focuses on podocyte injury in diabetes, the most common cause of acquired podocyte disease. In both human and rodent models of type 1 and type 2 diabetes (T1DM/T2DM (Umanath & Lewis, 2018)), clinical evidence of podocyte dysfunction—manifested as microalbuminuria (Kelly et al., 2002)—typically arises only after prolonged exposure to hyperperfusion, hyperfiltration, and metabolic stress (Wu et al., 2023). Identifying early structural alterations in podocytes prior to the onset of microalbuminuria can widen the currently understood window during which diabetic damage becomes detectable at the cellular level. This would greatly enhance our ability to investigate disease mechanisms and develop interventions aimed at halting the progression of diabetic nephropathy (DN), a condition that accounts for approximately one-quarter to one-third of patients reaching end-stage kidney disease in the United States (Umanath & Lewis, 2018).

Transmission electron microscopy (TEM) and standard light microscopy have traditionally been employed to assess the cross-sectional histological features of podocyte damage in DN (Conti et al., 2018). Under TEM, indices such as increased FP width (FPW) have been correlated with proteinuria in diabetic patients (White et al., 2004). However, TEM is inherently two-dimensional, providing only a limited view of podocyte ultrastructure (Fig. 1b). More recently, super-resolution microscopy techniques–including 3D structured illumination microscopy (SIM) (Siegerist et al., 2017; Artelt et al., 2018) and stimulated emission depletion (STED) (Unnersjo-Jess, 2023; Wiesner et al., 2024) –have been utilized to study SD organization by labeling nephrin with fluorescent markers. These approaches, while powerful, are limited by the need for specialized microscopes, extensive tissue processing, and strict sample preparation requirements.

In contrast, scanning electron microscopy (SEM) provides three-dimensional visualization of podocyte topography without the need for fluorescent labeling. Despite its advantages, scanning electron microscopy (SEM) remains underutilized for podocyte analysis due to the need for expert pathological interpretation and the absence of standardized quantitative metrics. To address this gap and establish SEM as a reliable, accessible tool for unbiased quantification, we developed and evaluated three novel image analysis methods using SEM images from an animal model of diabetic kidney injury, the streptozotocin (STZ)-induced diabetic mouse model.

## Methods

### STZ-induced Diabetic Nephropathy in C57BL/6J Mice

As described in a previous study (Wiesner et al., 2024), eight- week-old, fasted C57BL/6J male mice received daily intraperitoneal (IP) injections of 50 mg/kg body weight streptozotocin (STZ, MedChemExpress LLC, # HY-13753) dissolved in 0.1 M sodium citrate buffer (pH = 4.5) for 5 consecutive days (*n* = 15). Age and gender-matched vehicle control mice were injected with sodium citrate buffer alone, of the same volume and regimen (*n* = 17). Kidney, blood, and urine samples were collected 3 months after STZ injection for analysis. Whole blood glucose was measured from the tail vein blood with the Germaine^TM^ Laboratories AimStrip^TM^ Plus Blood Glucose Testing System. Urine was collected to measure the albumin and creatinine levels with the Albumin ELISA Quantitation kit (Bethyl Laboratories Inc.) and a colorimetric creatinine quantification kit (Bioassay Systems). The albumin to creatinine ratio (ACR) was calculated from these measurements.

### Kidney Histology and SEM Imaging

As described previously (Williquett et al., 2024), kidney cortex tissues were processed at the Central Microscopy Research Facility (CMRF) at the University of Iowa. Scanning electron microscopy (SEM) images were obtained at 12,000× magnification from glomeruli of mice grouped into two groups: non-diabetic and STZ-induced diabetic nephropathy (T1DM) cohorts. High-quality SEM images were acquired from 3–5 glomeruli per mouse. In total, 82 glomerular images were collected from 17 nondiabetic mice, and 73 glomerular images were collected from 15 STZ-induced diabetic mice.

### Data Acquisition With ImageJ

**Fig. 2.**
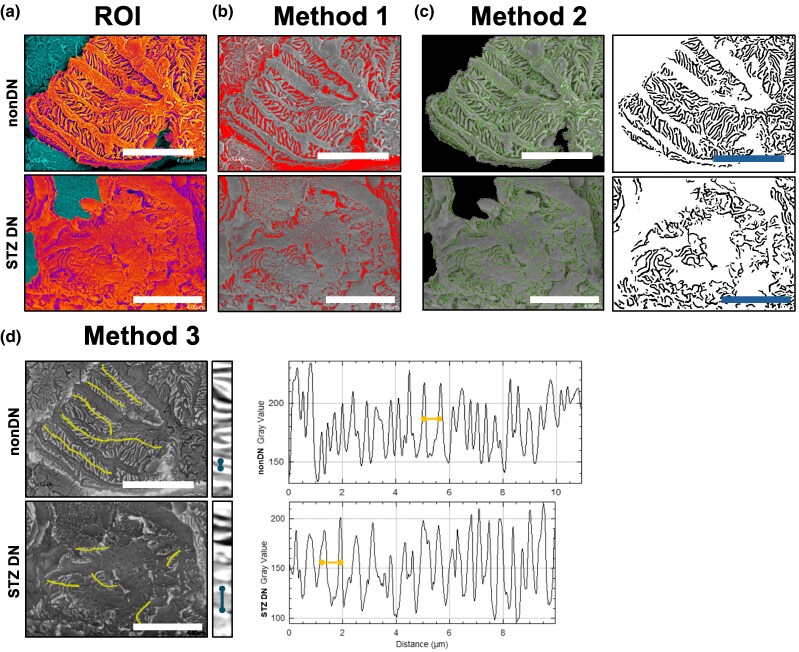
Methodology for quantification of slit diaphragm and foot process width using scanning electron microscopy (SEM). (**a**) SEM image with the region of interest (ROI) highlighted in orange. Scale bar = 4.00 *μ*m. (**b**) Slit diaphragm (SD) areas, highlighted in red, identified using an automated thresholding method. (**c**) Slit diaphragm–foot process ridges detected and highlighted in green. The corresponding binary images (right) represent the SD areas located between adjacent ridges. (**d**) Foot process (FP) profiles generated along yellow lines drawn perpendicular to multiple FPs. The straightened gray value profiles (right) were used to calculate foot process width (FPW) by measuring the distance (*μ*m) between adjacent peaks.

SEM images were analyzed using ImageJ software (NIH) with three complementary quantification methods focused on slit diaphragm (SD) and foot process (FP) morphometry. A region-of-interest (ROI) of good imaging quality was defined with the polygon selection tool (Fig. 2a).

### Method 1 Threshold-based SD Fraction Quantification

Here, the “*Accurate Gaussian Blur*” plugin (Schmid, 2008) (radius: 2.00) was applied to smooth ROI features before subsequent analyses, reducing artificial ridges caused by SEM-generated noise. The SD area was quantified in ImageJ using the thresholding tool with the “Yen” algorithm, which automatically segments the image by selecting an intensity threshold that maximizes the separation between foreground signal and background noise, thereby identifying regions corresponding to SDs (Fig. 2b, red area). The SD fractional area (SD fraction %) was calculated as the proportion of the ROI covered by the threshold-defined SD signal.

### Method 2 Ridge Detection for SD Area

In this case, the “*Accurate Gaussian Blur*” plugin (radius: 4.00) was applied to smooth ROI features prior to subsequent analyses. We then used the “Ridge Detection” plugin (Steger, 1998) of ImageJ (based on Steger's curvilinear detector) to identify ridges between the FPs and the SDs and generate a binary that allowed for a more precise definition of the area of SDs. The following plugin parameters were used consistently across all samples: sigma (scale) = 5.70, line width = 15 pixels, lower threshold = 0.00, upper threshold = 0.17, minimum line length = 5.00 pixels, and maximum line length = 0.00 pixels. The values of these parameters were determined by optimization on a representative training set of images to balance sensitivity and noise rejection (see Supplementary Methods and Supplementary Fig. 1) and then fixed for all experimental analyses. Then, the area defined by ridge detection (Fig. 2c, black area) over the ROI was quantified as the SD fraction%.

### Method 3 Foot Process (FP) Width via Plot Profile

For the final method, we quantified the foot process width as described in previous literature (Siegerist et al., 2017), using the “Plot Profile” feature of ImageJ. As delineated in Figure 2d, we generated a plot FP profile along a line perpendicular to multiple FPs, and the distance between the peaks was measured to determine the FP width. FP profiles of 3–5 contiguous regions per glomerulus were processed for calculation.

### Statistical Analysis

Data were analyzed using GraphPad Prism 10. Results are presented as mean ± SD for SD fraction (%) and FP width (*μ*m) per glomerulus. For both Method 1 and Method 2, the mean SD fraction (SD%) per mouse was calculated and used for comparisons between control and diabetic mice. For Method 3, the mean FP width per glomerulus was used for group comparisons (non-DN: *n* = 17; STZ-DN: *n* = 15). Unpaired two-tailed t-tests were used to compare non-diabetic and diabetic groups. Simple linear regression and nonlinear regression were applied to assess trends and linearity. Pearson correlation analyses were performed to assess the relationships of SD fraction (%) and FP width (*μ*m) with clinical markers like fasting glucose levels and microalbuminuria. Receiver Operating Characteristic (ROC) curves were generated to assess the sensitivity of SD fraction (%) and FP width as markers of differential podocytopathy between diabetic nephropathy (DN) mice and non-DN mice. A value of *p* < 0.05 was considered statistically significant.

### Reproducibility Analysis

To assess the reliability of the ridge detection-based slit diaphragm (SD%) measurements, a subset of 20 representative images (10 control, 10 diabetic) was randomly selected for reproducibility analysis. All images were analyzed blinded to the experimental group. Inter-rater reproducibility was assessed by having two independent raters measure SD% on all selected images. Intra-rater reproducibility was assessed by repeating measurements with one rater after an 8-week interval. For both analyses, Bland–Altman plots were generated in GraphPad Prism by plotting the mean SD% of each image on the *x*-axis and the difference between measurements on the *y*-axis. The mean difference (bias) and 95% limits of agreement (mean ± 1.96 SD) were used to quantify reproducibility.

### Ethics Statement

The animal protocol was approved by the Institutional Animal Care and Use Committee of the University of Iowa and is in accordance with the NIH guidelines for use of live animals. All work was performed in accordance with the principles and procedures outlined in the NIH guidelines.

## Results

### The Ridge Detection-based SD Fraction Is a Sensitive SEM Index for Diabetic Podocytopathy

**Fig. 3.**
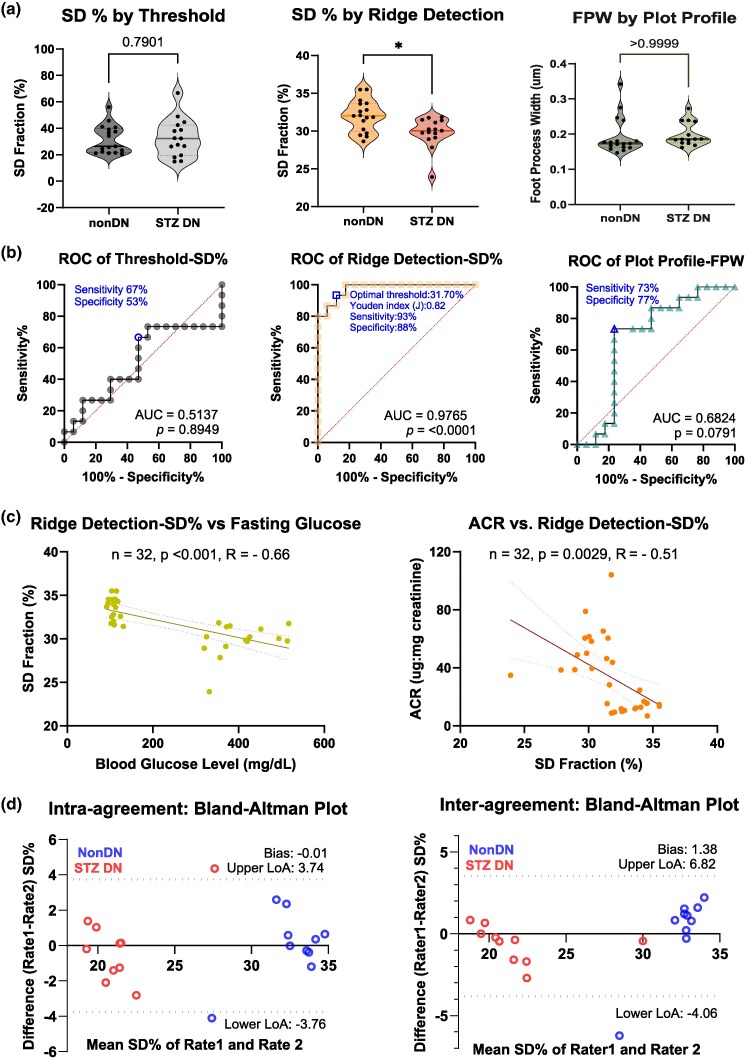
SEM-based quantification of podocyte ultrastructure. (**a**) Comparison of podocyte ultrastructural features between the glomeruli of nondiabetic (non-DN) mice and the glomeruli of mice with streptozotocin-induced diabetic nephropathy (STZ-DN). Violin plots display: (1) the average slit diaphragm (SD) fraction per mouse quantified by automated thresholding (Method 1), (2) the average SD fraction per mouse derived from the ridge detection (Method 2), and (3) the average foot process width (FPW) per mouse calculated from intensity profile plots (Method 3). non-DN: *n* = 17; STZ-DN: *n* = 15. *p < 0.05 (STZ-DN versus non-DN). (**b**) Receiver Operating Characteristic (ROC) curves evaluating the performance of the three SEM-derived indices in distinguishing diabetic podocytopathy, with corresponding statistical significance and area under the curve (AUC) values. non-DN: *n* = 17; STZ-DN: *n* = 15. (**c**) Pearson correlation analysis between the ridge detection–derived SD fraction and two diabetic nephropathy biochemical phenotypes, fasting blood glucose and albumin-to-creatinine ratio (ACR), using data from both diabetic and nondiabetic mice. *n* = 17 (non-DN) + 15 (STZ-DN) = 32. (**d**). Bland-Altman analysis of ridge-detection-based SD% measurements. The right panel shows the inter-rater agreement between Rater 1 and Rater 2, while the left panel shows the intra-rater agreement between two measurements by Rater 1, separated in time by 8 weeks. Each point represents an SEM image. Blue points indicate Non-DN values, and red points indicate STZ-DN values. The dashed lines represent the 95% limits of agreement. Most points fall within these limits, indicating good inter- and intra-rater reproducibility across both groups.

Three independent image quantification methods—the threshold-based SD fraction, the ridge detection-based SD fraction, and the plot profile-derived foot process (FP) width—were used to assess podocyte ultrastructural changes in STZ-induced diabetic mice and non-diabetic controls (Fig. 3a). The first two methods measure the fractional area of the glomerular region of interest occupied by slit diaphragms, while the third method measures the mean FP width per glomerulus (Figure. 1a).

Among these methods, only the ridge detection-derived SD fraction showed a statistically significant reduction in STZ-diabetic mice (STZ-DN, *n* = 15; mean ± SD = 29.82 ± 1.98%, 95% CI: 28.7–30.92%) compared to non-diabetic mice (non-DN, *n* = 17; mean ± SD = 32.00 ± 2.08%, 95% CI: 30.92–33.07%) (*p* < 0.05). The standardized mean difference (Hedges’ g) was 1.04 (95% CI: 0.32–1.77), reflecting both the sensitivity of this method and a large effect of diabetic injury on podocyte SD%. In contrast, no statistically significant differences were observed using the threshold-based SD fraction or the FP width measured by the plot profile method.

### The Ridge Detection-based SD Fraction Best Distinguishes Between Diabetic and Nondiabetic Features of Podocytes

Receiver Operating Characteristic (ROC) curve analysis was performed to evaluate the diagnostic performance of the three SEM quantification methods in distinguishing diabetic from non-diabetic podocyte morphology. The ridge detection-derived SD fraction demonstrated excellent discriminative ability, with an area under the curve (AUC) of 0.9765, indicating high diagnostic validity (Fig. 3b; *p* < 0.0001). Using the Youden index (0.82), the optimal SD% threshold was determined to be 31.70% (95% CI: 31.24–32.28%), corresponding to a sensitivity of 93% (95% CI: 70.18–99.66%) and a specificity of 88% (95% CI: 65.66–97.91%).

Bland-Altman analysis demonstrated minimal bias between repeated measurements, with a mean difference of −0.01 and 95% limits of agreement ranging from −3.76 to 3.74%, indicating excellent intra-rater reproducibility. Inter-rater reproducibility was similarly high, with a mean difference of 1.38 and 95% limits of agreement from −4.06 to 6.82% (Fig. 3d). Together, these results confirm the strong reproducibility of ridge-detection–based SD% measurements across both raters and repeated assessments.

In contrast, the threshold-derived SD fraction showed poor diagnostic accuracy (AUC = 0.5137, *p* > 0.05; sensitivity = 67%, specificity = 53%). The FPW by plot profile method yielded only moderate performance (AUC = 0.6824, *p* > 0.05; sensitivity = 73%, specificity = 77%). These data support the ridge detection method, which measures SD area fraction in ROIs, as a valid ultrastructural quantification approach for identifying diabetic podocytopathy.

### The Ridge Detection-Derived SD Fraction Correlates With Clinical Markers of Diabetic Nephropathy

To evaluate the clinical relevance of the SD fraction derived from ridge detection as an ultrastructural index of diabetic podocytopathy, Pearson correlation analyses were performed with fasting blood glucose (a major risk and causal factor for DN) and the albumin-to-creatinine ratio (ACR), a key clinical marker of DN. Scatter plots revealed significant negative correlations between SD fraction and fasting glucose levels (Fig. 3c; *n* = 32, *p* < 0.001, R = −0.66), as well as with ACR (*n* = 32, *p* = 0.0029, R = −0.51). These results indicate that the ridge detection–derived SD fraction is sensitive to diabetic stress (hyperglycemia) and correlates with glomerular barrier dysfunction as reflected by albuminuria.

## Discussion

From our data, we defined a novel ultrastructural index of SD fraction for identifying and quantifying features of diabetic podocytopathy. Specifically, the SD fraction quantified by the ridge detection method proved to be a valid and sensitive approach for capturing podocyte ultrastructural changes induced by diabetes. This method demonstrated strong diagnostic performance with high sensitivity, specificity, and reproducibility, and correlated well with established clinical indicators of diabetic kidney disease, including hyperglycemia and microalbuminuria.

Historically, TEM has been used to quantify podocyte foot process width (FPW) as an ultrastructural marker in both human biopsies and animal models of DN (Inoki et al., 2011; Li et al., 2025). As illustrated by our Method 3, which mimics TEM principles, FPW measurement was highly dependent on sectioning orientation and did not detect differences as effectively as ridge detection. Ridge detection enhances SEM power by incorporating topological quantification of the podocyte surface, often overlooked in conventional TEM analyses. Thus, our method offers an innovative tool capable of detecting and classifying subtle podocyte damage that precedes overt microalbuminuria, providing standardized metrics to evaluate disease severity and therapeutic efficacy. An additional strength of this method is its accessibility: it does not require specialized training in renal pathology and can be reliably applied by third-party analysts, potentially reducing observer bias.

However, several limitations remain. First, manual selection of regions of interest (ROIs) with adequate image quality is still required at the initial step, introducing potential user-dependent variability. Second, our analysis focused on only two indices—SD fraction and FP width—based on their hypothesized correlation with glomerular filtration barrier integrity and the development of albuminuria. To address these limitations, we plan to implement automated segmentation and quantification strategies using deep learning tools such as U-Net or Weka-based classifiers (Ronneberger et al., 2015; Arganda-Carreras et al., 2017), which enable automated boundary detection, potentially eliminating the need for manual selection of ROIs. These approaches will enhance reproducibility, reduce user bias, and improve scalability for broader applications in both research and clinical settings. These AI tools also allow the incorporation of additional topological parameters, including FP/SD regularity, spacing, and size distribution, for a more comprehensive assessment of podocyte morphology.

While our SEM analyses offer superior resolution for visualizing podocyte surface architecture, they do not allow direct imaging or measurement of the glomerular basement membrane (GBM) or the fenestrated endothelial cells beneath the GBM, which also undergo pathological changes in diabetic nephropathy (Qi et al., 2017). Future work integrating SEM-derived indices with TEM-based 2D-measures —such as FPW, GBM thickness, podocyte density, and endothelial changes—as well as automated image analysis with 3D reconstruction or super-resolution immunofluorescence imaging (Unnersjo-Jess, 2023) will define the full spectrum of diabetic glomerulopathy. Although TEM has been more widely used for clinical diagnosis in human kidney biopsy specimens, SEM has also been successfully applied, providing a more sensitive and direct view of diabetic podocyte injury at very early stages (Conti et al., 2018). Thus, the timely development of SEM-based ultrastructural indices has the potential to improve pathological diagnostics and enhance clinical practice.

Finally, our initial analyses were performed in an STZ-induced diabetic model developed in C57BL/6J mice, which are relatively resistant to glomerular disease (Breyer et al., 2005; Qi et al., 2005, 2017; Wang et al., 2023). Male mice were chosen due to the known resistance of females to diabetic stress. This approach allowed us to establish a sensitive SEM-based metric in a genetically uniform background compatible with our other transgenic podocyte-specific models. Nonetheless, our future validation will extend this method to DKD-susceptible strains (e.g., DBA/2), additional models, and stages of DN (e.g., db/db and eNOS −/− ; db/db mice) in both sexes. Application to other podocytopathies with distinct ultrastructural features will further test the generalizability of this approach, and cross-validation by independent groups will ensure reproducibility and applicability.

## Concluding Remarks

We established a standardized scanning electron microscopy-based pipeline to quantify podocyte slit diaphragm (SD) fraction as an ultrastructural index reflecting podocyte function. Specifically, a ridge detection–based SD fraction demonstrated a high diagnostic performance for distinguishing diabetic from nondiabetic podocytes. This index enables accurate detection of early podocyte injury in diabetes, providing a valuable tool for future mechanistic and therapeutic studies.

## Availability of Data and Materials

All original data in the manuscript are available. There are no human clinical, genetic, or omics data to declare in our article.

## Supplementary Material

ozaf122_Supplementary_Data

## Supplementary Material

To view Supplementary material for this article, please visit https://doi.org/10.1093/mam/ozaf122.
