# Supplementary material for: A New Quantitative Metric for Precise Classification of Diabetic Podocyte Injury Using Scanning Electron Microscopy
Source: Microsc Microanal. 2025 Dec 10;31(6):ozaf122. doi: 10.1093/mam/ozaf122 (PMC12693405; doi:10.1093/mam/ozaf122)
Supplement: ozaf122_Supplementary_Data [file ozaf122_supplementary_data.pdf]

Supplementary Materials

- Supplementary Methods
- Supplementary Figure 1

Supplementary Methods

Podocyte Ridge Detection Pipeline: This pipeline uses ImageJ/Fiji for quantifying podocyte slit diaphragm and foot process features in SEM images. The pipeline is based on the Ridge Detection plugin (Steger's curvilinear detector) with standardized preprocessing and parameter settings to ensure reproducibility across samples.

1. Input: SEM images of podocytes in TIFF format.
2. Preprocessing:
   - Select the region-of-interest (ROI) and clear outside.
   - Apply Gaussian blur (σ = 4.0 pixels)
3. Ridge detection (ImageJ Ridge Detection plugin) with parameters set to:
   - Sigma (scale): 5.70
   - Line width: 15.0 pixels
   - Lower threshold: 0.00
   - Upper threshold: 0.17
   - Minimum line length: 5.00 pixels
   - Maximum line length: 0.00 pixels
   - Check "Darkline", "Estimate Width", "Extend line" and "Make Binary"
4. Output:
   - Overlay the Binary Ridge Map with the region-of-interest (ROI) and calculated ridge density (SD%) per image.

Optimization of Podocyte Ridge Detection Parameters: Parameters were iteratively optimized on representative images to balance sensitivity (minimizing false negatives) and noise rejection (minimizing false positives). A range of sigma values and threshold levels (±50% of the selected values) was tested, with representative non-DN and DN images illustrating the rationale for parameter choice (Supplementary Figure 1).

Supplementary Figure 1

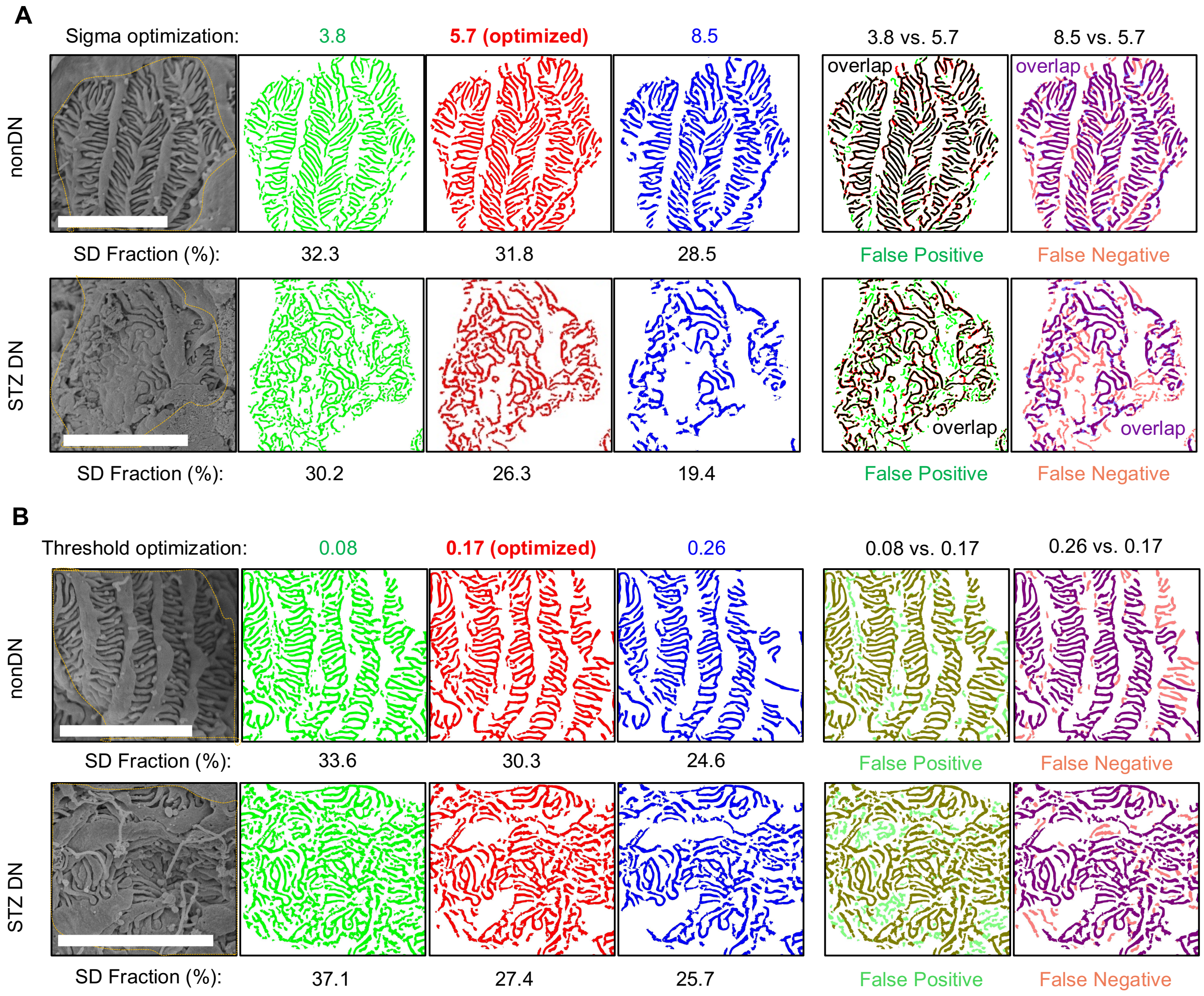

Supplementary Figure 1. Optimization of podocyte ridge detection parameters.

(A) Sigma parameter training. The slit diaphragm (SD) pattern detected with the optimized sigma value of 5.7 was compared with those obtained using sigma values of 3.8 and 8.5. Relative to the optimized setting, sigma = 3.8 generated more false-positive artifacts (highlighted in green; 3.8 vs. 5.7), whereas sigma = 8.5 failed to detect certain SD regions, resulting in false negatives (highlighted in red; 8.5 vs. 5.7).

(B) Upper threshold parameter training. The SD detected with the optimized threshold of 0.17 was compared with those obtained using thresholds of 0.08 and 0.26. Threshold = 0.08 generated more false-positive artifacts (highlighted in green; 0.08 vs. 0.17), while threshold = 0.26 failed to detect some SD regions, leading to false negatives (highlighted in red; 0.26 vs. 0.17).

Scale bar = 4.00 μm.
